# A Dictionary of Practical Medicine

**Published:** 1834-01-01

**Authors:** 


*A Dictionary of Practical Medicine.* By JAMES COPLAND, M.D. Parts I. and II.—London, 1833. 8vo. pp. 640 and xxxii.

A COMPILER who has not only taste enough to select the best fragments of a thousand works, but spirit enough to connect them into one harmonious whole, and thus to breathe Promethean fire into those lifeless particles, is one of the rarest phenomena of the literary world; yet such a compiler is Dr. Copland. The parts now before us comprise about eighty articles, from Abdomen to Encysted Dropsy, and not only contain the essence of whole libraries, but are replete with observations which could come from none but a practical physician. We do not think it necessary to give any extracts in this place, as the reader will find several in our Collectanea.

